# Application of Nanomedicine in Inner Ear Diseases

**DOI:** 10.3389/fbioe.2021.809443

**Published:** 2022-02-11

**Authors:** Qianyu Lin, Qiong Guo, Mingchao Zhu, Juanli Zhang, Bei Chen, Tingting Wu, Wei Jiang, Wenxue Tang

**Affiliations:** ^1^ Department of Molecular Pathology, Application Center for Precision Medicine, The Second Affiliated Hospital of Zhengzhou University, Zhengzhou, China; ^2^ Center for Precision Medicine, Academy of Medical Sciences, Zhengzhou University, Zhengzhou, China; ^3^ State Key Laboratory of Natural Medicines, Jiangsu Key Laboratory of Drug Discovery for Metabolic Diseases, Jiangsu Key Laboratory of Drug Screening, China Pharmaceutical University, Nanjing, China; ^4^ Henan Institute of Medical Device Inspection, Zhengzhou, China; ^5^ Department of Otology, the First Affiliated Hospital of Zhengzhou University, Zhengzhou, China

**Keywords:** inner ear disease, nano-based drug delivery, local therapy, specific delivery, blood–inner ear barrier

## Abstract

The treatment of inner ear disorders always remains a challenge for researchers. The presence of various physiological barriers, primarily the blood–labyrinth barrier (BLB), limits the accessibility of the inner ear and hinders the efficacy of various drug therapies. Yet despite recent advances in the cochlea for repair and regeneration, there are currently no pharmacological or biological interventions for hearing loss. Current research focuses on the localized drug-, gene-, and cell-based therapies. Drug delivery based on nanotechnology represents an innovative strategy to improve inner ear treatments. Materials with specific nanostructures not only exhibit a unique ability to encapsulate and transport therapeutics to the inner ear but also endow specific targeting properties to auditory hair cells as well as the stabilization and sustained drug release. Along with this, some alternative routes, like intratympanic drug delivery, can also offer a better means to access the inner ear without exposure to the BLB. This review discusses a variety of nano-based drug delivery systems to the ear for treating inner ear diseases. The main factors affecting the curative efficacy of nanomaterials are also discussed. With a deeper understanding of the link between these crucial factors and the clinical effect of nanomaterials, it paves the way for the optimization of the therapeutic activity of nanocarriers.

## Introduction

The most recent global report suggests that over 1.5 billion people were experiencing some degree of hearing loss in 2021. This estimate is projected to rise to over 2.5 billion by 2050 (WHO, 2021). Many dysfunctions of the inner ear, such as sudden sensorineural hearing loss (SNHL), noise-induced hearing loss, and autoimmune inner ear disease, are usually the preludes to deafness (Pritz et al., 2013). The inner ear is surrounded by the hardest bone in the human body, and its limited accessibility, narrow space, and high susceptibility limit the choice of drug types and dosage forms for delivery to the inner ear (Poe and Pyykko, 2011). Furthermore, the limited vascular blood flow supply of the round window membrane (RWM), the protective inner ear blood barrier, and the low permeability of the round window membrane present great challenges for drugs to pass through the RWM into the cochlea. The pathogenesis of many causes of inner ear diseases occurs in a specific site in the inner ear, such as spiral ganglion neurons (SGNs), cochlear nerve, or sensory hair cells, and it would be ideal to apply the therapeutic agent only to those affected cells (Roy et al., 2010; Zhang et al., 2012; Wang et al., 2014; Yoon et al., 2016; Kayyali et al., 2018).

The therapeutic agents that are under consideration for inner ear delivery can be divided into five groups: (1) antibiotics, (2) anti-inflammatory agents, (3) antioxidants, (4) neurotrophins, and (5) genetic material. Up to now, conventional drug therapy has been considered to be the primary strategy for the treatment of hearing loss. However, it should be noted that inner ear hearing loss here generally refers to SNHL. Treatment of conductive hearing loss, which arises from the ear canal or middle ear, consists of either rehabilitation with hearing aids or performing surgery. In contrast to drug therapy, gene therapy promises to be developed as a treatment targeting causal genes for genetic deafness. While there are still many limitations that will lead to the reduced efficacy of genetic materials in clinical use, such as short half-life, infant technology, and serious safety concerns, the need to cope with an increasing number of diverse therapeutics for the otological field has led to the application of the delivery systems to the ear. Most frequently, these carrier materials are applied as nanoparticles (NPs). In the past decade, many NP (also called nanocarriers or nanovectors) systems have been developed for inner ear drug delivery, including polylactic/glycolic acid (PLGA) NPs (Horie et al., 2010b), magnetic NPs (Liang et al., 2020), lipid NPs (Meyer et al., 2012), and polymersomes (Roy et al., 2010).

The first basic step in the design of drug administration protocol is to determine the optimal route and formulation. The clinical therapy to date has consisted mostly of the traditional cochlea plant, systemic administration of steroids (Iwasaki et al., 2017), and local drug delivery routes. The intravascular route is an attractive, non-invasive strategy to avoid the otic capsule and inaccessible anatomy surgical procedures, while localized drug delivery systems are now being increasingly used and improved the technologies. Local administration has been shown to successfully deliver biomaterials into the inner ear *via* the RWM due to reducing the adverse effects of the above-mentioned drug delivery (Valente et al., 2017). Certain classes of medicine carriers exhibit additional characteristics that offer other unique preventive and diagnostic advantages combined with local application to the inner ear. The nanocarriers which possess smaller particle size, amphiphilic behavior, high drug loading, and controlled and sustained release protect the drug from the surrounding environments and reduce the dosing frequency. On the other hand, due to the smaller size and surface charge, some defects in NPs, such as poor drug delivery capacity, poor stability, and poor accumulation in healthy organs, are unavoidable (Odiba et al., 2017). Therefore, all factors should be considered when designing nanocarrier-based formulations.

**FIGURE 1:**
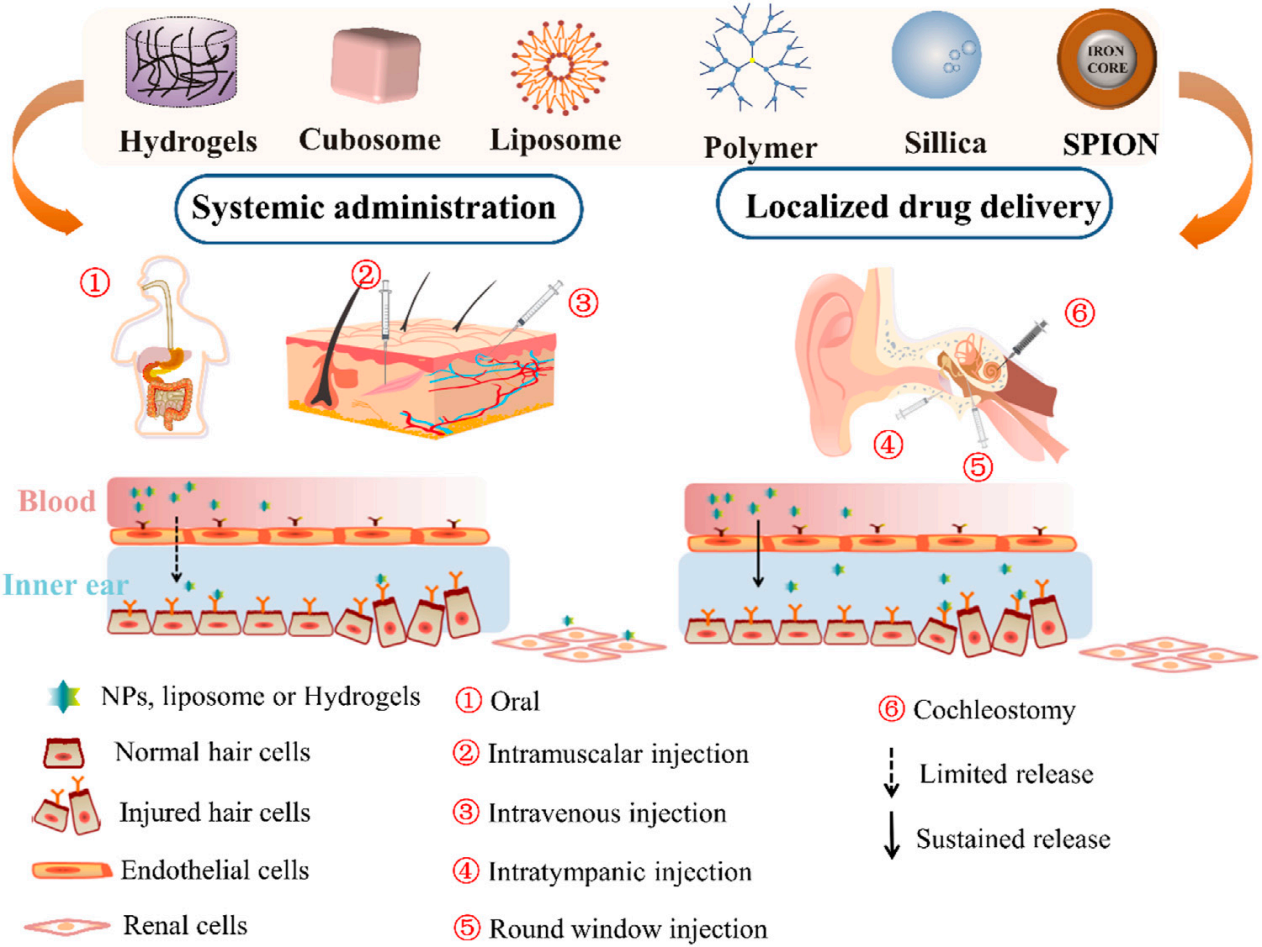
Scheme of the general administration route of nanomaterials, including hydrogels, cubosome nanoparticles (NP), liposomes, polymer NP, silica NP, and SPION for inner ear therapy. SPION, supermagnetic iron oxide nanoparticle.

In this review, we will summarize the barriers to inner ear drug delivery, specific sites within the inner ear, and administration route. Furthermore, the present review aims to provide an updated general overview of nano-based delivery approach and factors that affect the delivery of nano-sized materials to the inner ear (Figure 1).

## The Barriers to Inner Ear Drug Delivery

The human inner ear is separated from the external system and is protected from any intense stimuli, pathogens, and foreign matters *via* various physiological barriers. These barriers maintain the homeostasis of the ear and regulate the entryway of essential nutrients, ions, proteins, and metabolites inside and outside the ear. However, for drug delivery, these physiological barriers may be the main factors that prevent the drugs from entering the inner ear.

Though systemic drug delivery allows drugs to enter the inner ear intravascularly through the blood vessels, low local blood supply often results in subtherapeutic local concentrations. The drugs likely travel through the labyrinthine artery, which branches into the spiral modiolar artery, followed by the vestibulocochlear artery. The spiral modiolar artery supplies blood into the stria vascularis and organ of Corti and allows drugs to enter into these regions (Rathnam et al., 2019). Furthermore, ear diseases lead to changes in capillary permeability and a decrease in blood flow. Besides these, excessive production of reactive oxygen species (ROS) can also lead to a decreased blood flow rate in the inner ear. ROS-induced cochlear damage may be caused by noise exposure, and ROS can oxidize lipids to form vasoactive lipid peroxidation molecules, like isoprostanes (Ohinata et al., 2000). These toxic products may reduce the cochlear blood flow.

The BLB always limits the accessibility of the inner ear and hinders the efficacy of various drug therapies. Similar to the blood–brain barrier that separates brain interstitial fluid from the blood, it covers all of the primary anatomical barrier structures separating cochlear (and vestibular) fluids and tissues from the blood (Nyberg et al., 2019). The barrier consists of non-fenestrated capillaries with a continuous endothelial lining, with tight and adherent junctions between endothelial cells. The tight junction preferentially excludes many high-molecular-weight compounds from the blood as well as most agents and restricts their entry into the inner ear tissues. To avoid BLB-restricted systemic delivery into the inner ear, local administration and targeting ligands to bind receptors on the surface of the inner ear cells, particularly to the damaged cells by circumventing the physiological barriers, is developed as a promising therapy for the treatment of ear disorders.

**FIGURE 2:**
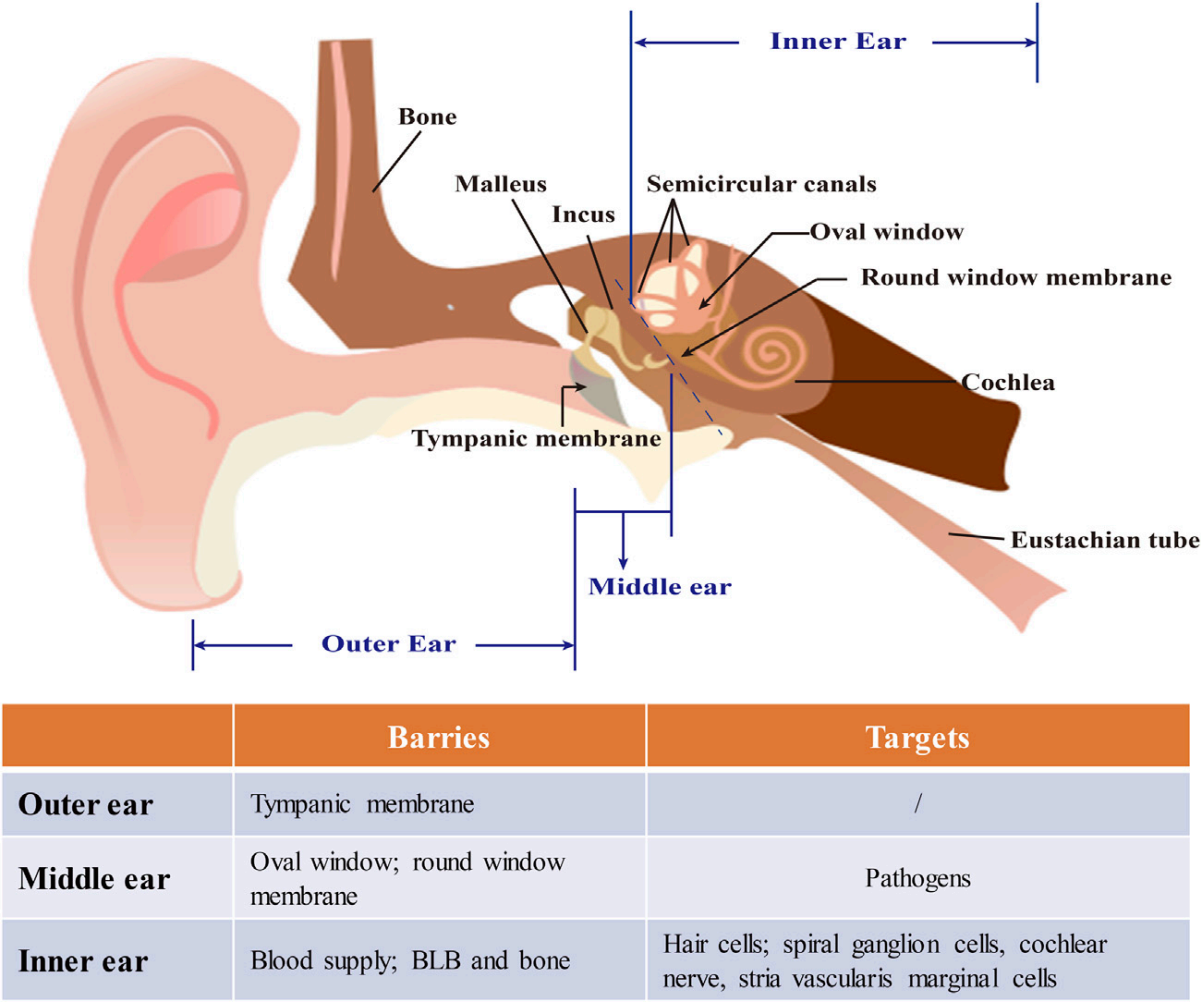
The structure of the ear and barriers and targets in nanomedicine-based treatment of inner ear diseases. The ear is composed of outer, middle, and inner ears. The tympanic membrane which separates the outer from the middle ear anatomically is a major barrier for drug delivery and can be bypassed by minimally invasive approaches like an intratympanic injection. The middle ear face of the round window membrane (RWM) is the pathway for noninvasive nanomedical drug delivery to the inner ear. Nanocarriers are usually locally loaded onto the RWM in gel for a sustained release of drugs. The inner ear is thought rarely to be a target for operations indirect drug delivery because of the risk of infection. Due to the characteristics of inner ear diseases, neurons and hair cells are often the main targets in its nanomedicine-based treatment.

The current inner ear drug delivery strategies rely on another connection between the middle and inner ears, the RWM, which is composed of three layers—an outer epithelium, a middle core of connective tissue, and an inner epithelium. The layers of the RWM play a role in participating in the absorption and secretion of substances within the inner ear (Goycoolea and Lundman, 1997). The passage of substances through the RWM is by different pathways, the nature of which is seemingly determined by the outer epithelium of the RWM. As a result, therapeutic agents fail to reach the targeted cells even in an effective concentration due to the effective connecting pore size of the outermost epithelial layer being only 0.6 nm (Spring 1998) **(** Figure 2).

## Targets for Drug Delivery

In most cases, the pathogenesis of auditory impairment occurs in specific cell types in the inner ear, such as SGNs, sensory hair cells, supporting cells, stria vascularis marginal cells, and spiral ligament fibrocytes. Because hair cells cannot regenerate in the mammalian cochlea, cochlear damage caused by noise and ototoxic drugs may result in severe deafness if large numbers of hair cells are lost. While hair cells are innervated by the neurons of the cochlear ganglion, cochlear neurons may degenerate secondarily after the loss of inner hair cells (IHCs). The damage tends to accumulate, leading to profound deafness. It suggests that those traumatized cells should be an ideal target to apply therapeutic materials (Pinyon et al., 2014). At the pre-clinical level, there is mounting evidence of the therapeutic protection and/or regeneration of hair cells, SGNs, and other affected cell types with numerous examples of hearing protection and hearing recovery. In addition to immediate damage, apoptosis associated with oxidative stress has been implicated in the pathogenesis of hair cells and hearing loss. One of the signaling cascades that mediates apoptotic death is the c-Jun-N-terminal kinase (JNK) pathway, also known as the stress-activated protein kinase. The application of anti-apoptosis agents, such as JNK inhibitors or caspase inhibitors, has been demonstrated to reduce outer hair cell (OHC) death following an acute insult (Pirvola et al., 2000; Wang et al., 2007).

## Therapy for Inner Ear Diseases

Due to these specificities of the inner ear, many drugs have been developed to defend against inner ear diseases. The anti-inflammatory agents, such as glucocorticosteroid and dexamethasone, are designed to prevent, decrease, or reverse the inflammatory response to inner ear hair cell and nerve cells (Trune and Canlon, 2012). Different types of antibiotics and aminoglycosides focus on the inhibition of recurring bacterial infections and severe Meniere’s disease (Hesse et al., 2013). For hair cell damage caused by ROS, antioxidants are being considered to counteract the deleterious effects of ROS and may be effective in the prevention or treatment of oxidative stress-related diseases, including noise-induced SNHL (Pak et al., 2020). Brain-derived neurotrophin factor (BDNF) and their receptors are the key factors in stimulating cochlear neuron growth, increasing SGN survival after ototoxic damage and regenerating inner ear hair cell-like hair cells under proper conditions (Johnson Chacko et al., 2017). However, several compounds have shown a protective or rescuing effect against hearing loss in clinical studies but do not in practice because of the inappropriate strategies for inner ear drug delivery.

Systemic drug delivery includes intravenous, intramuscular, and oral routes. It has been successfully used in the clinic to treat vertigo (Fowler, 1948), SNHL, noise hearing loss, and Meniere’s disease. The first application for controlling inner ear diseases included systemic steroids for sudden SNHL and vestibular neuritis. The subsequent efficient local drug delivery to the inner ear was transformed from the initial systemic drug delivery (McCall et al., 2010). There is evidence that systemic delivery shows a limited bioavailability in results because of the rapid clearance from the circulation in the liver and spleen (Horie et al., 2010a). However, because there is no continuous passage through the BLB at very high doses, only a small part of the drug dose reaches the inner ear, resulting in many undesirable off-target side effects. These side effects may not relieve hearing loss but worsen it, leading to patients being less compliant and to organ failure or even death in severe cases. Consequently, another approach-localized drug delivery appears more suitable for the inner ear.

Compared with traditional systemic drug delivery, local tympanic drug delivery strategies could be more efficacious and can reduce systemic side effects. Intratympanic therapy is appropriate for low-molecular-weight molecules that can pass through the oval or round windows and the drugs that lack selectivity in their sites of action. It allows the drug to enter the inner ear from the middle ear, avoiding the labyrinthine artery and the blood–inner ear barrier, and the concentrations of the medicine in the perilymph and endolymph in the inner ear are higher than that of oral administration (Li et al., 2017), therefore making it easier to reach the effective concentrations for efficacious treatment. As early as 60 years ago, administering therapeutics *via* intratympanic injections was used for Meniere’s disease (Schuknecht, 1956). Subsequently, in 1991, it was first reported that Meniere’s disease was treated by successfully utilizing an intratympanic injection of dexamethasone (Itoh and Sakata, 1991). Since then, as a preferred drug administration strategy, it has been applied for delivering drugs into the inner ear to treat a variety of inner ear diseases, such as sudden SNHL. At present, middle ear injection is widely used in the clinic by direct needle injection or tympanostomy, whereas if the drug administration is not for one time but repeated and continuous, there may be a risk of middle ear infection, even if it is only minimally invasive (Licameli et al., 2008). A locally administered intratympanic agent represents an exciting strategy to improve inner ear treatments.

This efficacious drug delivery through the round or oval window membrane avoids potential inner ear damage caused by cochleostomy. Among the established routes for direct local administration, the two injection routes *via* the RWM—RWM injection and RWM injection with semi-circular canal fenestration—enable the vector to be delivered into the perilymph, which is clinically feasible (Yoshimura et al., 2021). The oval window membrane, the oval semi-permeable membrane on the middle eardrum, connects with the vestibule of the labyrinth of the inner ear and is covered by the bottom plate of the stapes and the surrounding circular ligament. One study by Rathnam *et al.* showed that the oval window membrane is more permeable than the RWM, and administration to the inner ear through the oval window membrane may be a promising way to treat vestibular dysfunction (Rathnam et al., 2019). Minimally invasive drug delivery across the round or oval window membrane was also implemented in the form of hydrogel, which more consistently releases therapeutics. Highly variable pharmacokinetic parameters are often observed in drug delivery across the human RWM. The exact quantification is difficult to study, in a large part due to the risk of permanent hearing loss when sampling inner ear fluids. These findings indicate that the targetability of specific peptides or nucleic acid will be improved by integrating local administration to the SGN cell population of the cochlea.

When a liquid dosage form is injected into the middle ear cavity, the drug may not be able to contact the RWM and be washed away by the Eustachian tube and flow to the nasopharynx, which further restricts the drug from entering into the inner ear. To overcome the drug loss of the middle ear, intracochlear drug administration allows the drug to enter the inner ear directly. Several methods provide direct access to the cochlea, that is, cochleostomy followed by direct injection, osmotic pump, and cochlear prosthesis-mediated delivery (Ayoob and Borenstein, 2015). Nevertheless, clinicians generally avoid employing intracochlear delivery directly as it requires traumatic surgery, which may cause infection or even worsening of the pathological environment of the cochlea. The intracochlear administration route was also used to minimize the off-target effects, but its high invasion and limitation to surgery cases are the major shortcomings in widespread clinical application. Newer approaches for localized drug delivery into the inner ear have considered these limitations and employed novel biomaterials to prolong drug exposure to targeted cells with minimal invasion.

**FIGURE 3:**
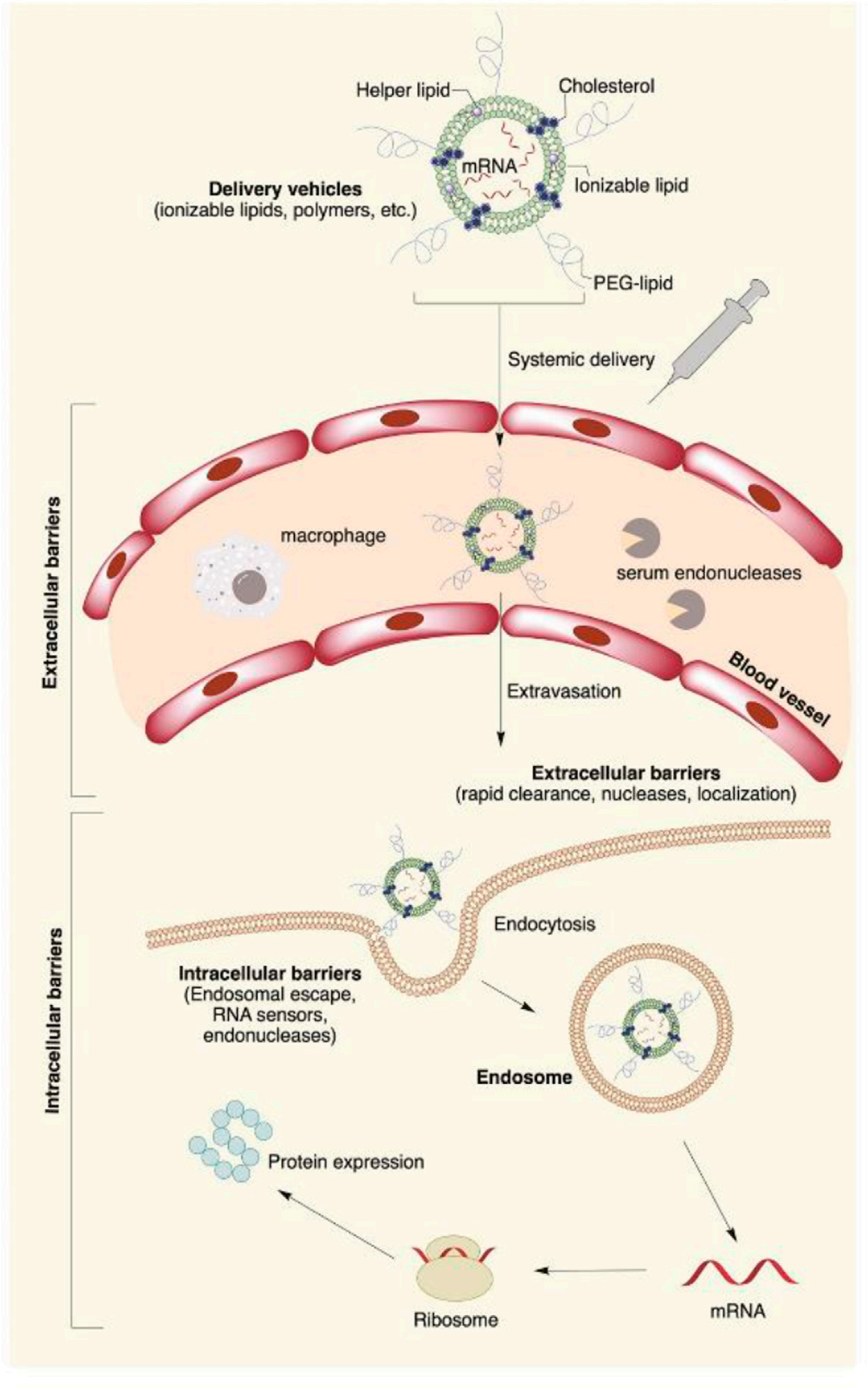
Main extra- and intracellular barriers for nanocarrier-mediated mRNA delivery. The negative potential across the cell membrane creates a major barrier for highly negatively charged mRNA molecules. The cell membrane is also a potential target for nanomedical drug delivery. Apart from the cell membrane, mRNA faces degradation by extracellular ribonucleases abundantly present in the skin and blood. Once inside the cell, lipid nanoparticles are routed into endosomes, followed by the lysosomes, where the mRNA contents are enzymatically degraded. Reproduced from van den Berg et al. (2021) with permission under the CC BYNC-ND 4.0 license (https://creativecommons.org/licenses/by-nc-nd/4.0/legalcode).

Recent developments in the field of gene therapy are showing exciting trends towards clinical therapies for hearing loss, particularly for the repair of genetic defects that result in the loss of hair cells (Izumikawa et al., 2005). Congenital hearing loss is a relatively common disorder, occurring in 1 to 2 per 1,000 newborns, among which 60–70% of cases are responsible for intricate genetic causes (Morton and Nance, 2006). The combination of repair of genetic defects includes gene replacement, gene augmentation, gene editing, post-translational modifications, and alternative splicing (Ma et al., 2019). RNA interference can be used to silence specific genes linked to protect against hearing loss, particularly for gain-of-function mutations—for instance, *GJB2*, encoding gap-junctional protein connexin-26 (Cx26), is responsible for the majority of genetic hearing loss in the connexin gene family, particularly non-syndromic hearing loss in most populations (Wingard and Zhao, 2015). A point mutation in connexin-26 results in a protein that interferes with the function of gap junctions, preventing hearing transduction in the cochlea through deficient potassium recycling (Cohen-Salmon et al., 2002). To suppress the mutant protein, a GJB2-targeting short-interfering RNA was complexed to a liposome delivery system and applied to the intact round window membrane of a mouse model of the connexin point mutation (Maeda et al., 2005). The RNA interference treatment significantly and specifically suppressed the mutant GJB mRNA without affecting the level of endogenous murine *Gjb2* expression, preventing hearing loss in the mouse model. More than 50% of mutations in Usher syndrome are in the *USH2A* gene. The large size of the *USH2A* gene, up to 15 kb, makes it difficult to apply a conventional gene therapy, but the CRISPR/Cas9 gene editing technology now offers an alternative strategy. The CRISPR/Cas9 system was employed in the study for *USH2A* gene manipulation, repairing the most prevalent mutations c.2299delG and c.2276G > T in exon 13 of *USH2A* (Fuster-Garcia et al., 2017). As the potential for drug and gene to restore hearing becomes evident in pre-clinical studies, the success of drug therapy and gene therapy also depend on the delivery vector to transfer the compounds specifically to target cells and the effective and durable expression in target cells (Figure 3).

## Nanocarrier System for Drug Delivery

**FIGURE 4:**
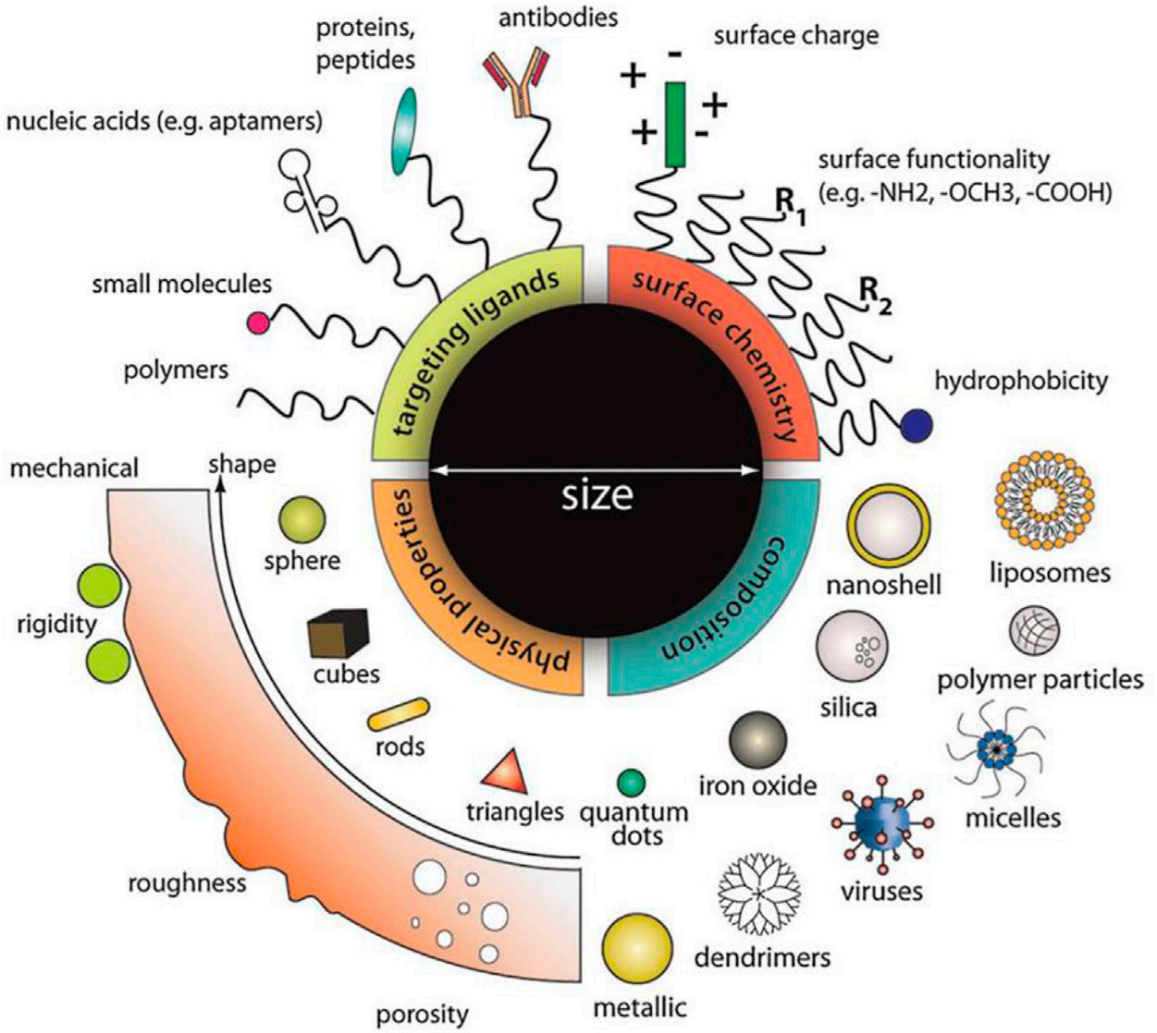
Designing multifunction nanoparticles for ideal treatment requirement. Material composition, surface properties, and molecule size can all be considered to optimize the functional properties of nanoparticles, including drug loading, release kinetics, cellular uptake, biological stability, and many more. Reprinted with permission from Chou et al. (2011).

The nano-sized particles, ranging from tens to several hundreds of nanometer in diameter, and nanotechnology are rapidly sweeping through all the vital fields of science and technology, such as electronics, aerospace, defense, medicine, and catalysis (Li et al., 2021). The nanocarriers employed in nanomedicine are made of less toxic materials, including but not limited to synthetic biodegradable lipids, polymers, and silica. The incomparable advantages of nanomaterials make it a new approach for the clinical treatment of hearing loss (Figure 4).

### Specific Targeting Property

The main problems currently associated with systemic drug administration include the distribution of therapeutic drugs, the lack of drug specificity towards a pathological site, nonspecific toxicity, and other adverse side effects. Current attempts to solve these problems are focusing on the localized use of targeted NPs. Magnetic targeting using superparamagnetic iron oxide nanoparticles (SPIONs) is one of the principal schemes to achieve this goal for the continuous maintenance of drugs.

SPIONs consist of cores made of iron oxide magnetite (Fe_3_O_4_) and/or maghemite (Fe_2_O_3_) that can be targeted to the required area through external magnets. They show interesting properties, such as superparamagnetism, high field irreversibility, high saturation field, extra anisotropy contributions, or shifted loops after field cooling. Due to these properties, the particles no longer show a magnetic interaction when the external magnetic field is removed (Mahmoudi et al., 2011).

SPIONs encapsulating corticosteroid was reported to protect hair cells against 4-hydroxynonenal-induced hair cell damage (Wang et al., 2011). Nanocarriers might be delivered to tissues with increased accuracy and efficacy by external magnetic targeting, which represents a promising way to deliver corticosteroids to the inner ear and to release such therapeutics locally in a controlled and sustained manner.

Similarly, the intratympanic injection of NPs containing prednisolone with an external magnetic field was applied to control the magnetic NP transportation to the inner ear and released with a clinical therapeutic dose, which alleviated the increase of high-frequency hearing threshold and the injury of cochlear OHCs induced by cisplatin (Ramaswamy et al., 2017).

Compared to intratympanic administration, a magnetic field can be applied for better delivery of therapy directly to the cochlea and can confer stronger diagnostic and therapeutic effects. Magnetic NPs loaded with prednisolone showed a significant reduction of hearing loss and increased outer hair cell survival in cisplatin-treated mice using a magnetic field (Ramaswamy et al., 2017).

Other nanomaterials with specific targeting properties are derived from a surface modification design. It is reported that the nanogel combined with a segment of polypeptide PrTP1 protects the ear from noise damage by targeting the motor protein prestin, which is expressed on the membrane of cochlear OHCs (Zheng et al., 2000). To reduce the hearing loss caused by acoustic damage, the targeted nano-based delivery system is applied to transport the JNK inhibitor, D-JNKi-1, in the Jun channel to the OHCs at the round window niche (Kayyali et al., 2018).

Surface charge and hydrophilicity in lipid-based NPs can change the uptake and targeting. Such modified liposome-targeted cell surface expressing glycoproteins, by using positively charged particles to promote cellular uptake in the mouse RWM, provided a protective anti-inflammatory effect from kanamycin and furosemide treatment (Yang et al., 2018). The addition of cationic charge and increase of hydrophilicity of dexamethasone-based NP can potentially be used for inner ear delivery if cytotoxicity is limited.

Various amounts of bioconjugation techniques, including maleimide (Kostiv et al., 2020), N-hydroxysuccinimide (Wilhelm et al., 2013), and carbodiimide-based (Escareno et al., 2021) chemistries, are used to add specific ligands onto the NP surface, although there has been limited research on targeted NP delivery into the inner ear.

### Drug Loading Capability

Notably, it is impossible for some novel drugs that are prone to inactivation, such as peptide, aptamer, and other genetic materials like DNA, mRNA, or siRNA, to rely on pure drug molecules without loss of therapeutic function. In contrast, nanocarriers have the capacity to stabilize their properties and are applicable for delivering a wide variety of therapeutic agents in different inner ear diseases. The unique advantages of lipid vesicles are their diverse range of morphologies, compositions, and abilities to envelope and protect many types of therapeutic biomolecules without an immunogenic response. Cationic liposomes can bind to negatively charged DNA molecules to form lipoplexes and be used for gene delivery. It is worth mentioning that the first successful gene delivery to the cochlea of a guinea pig was demonstrated by cationic liposomes (Wareing et al., 1999).

**FIGURE 5:**
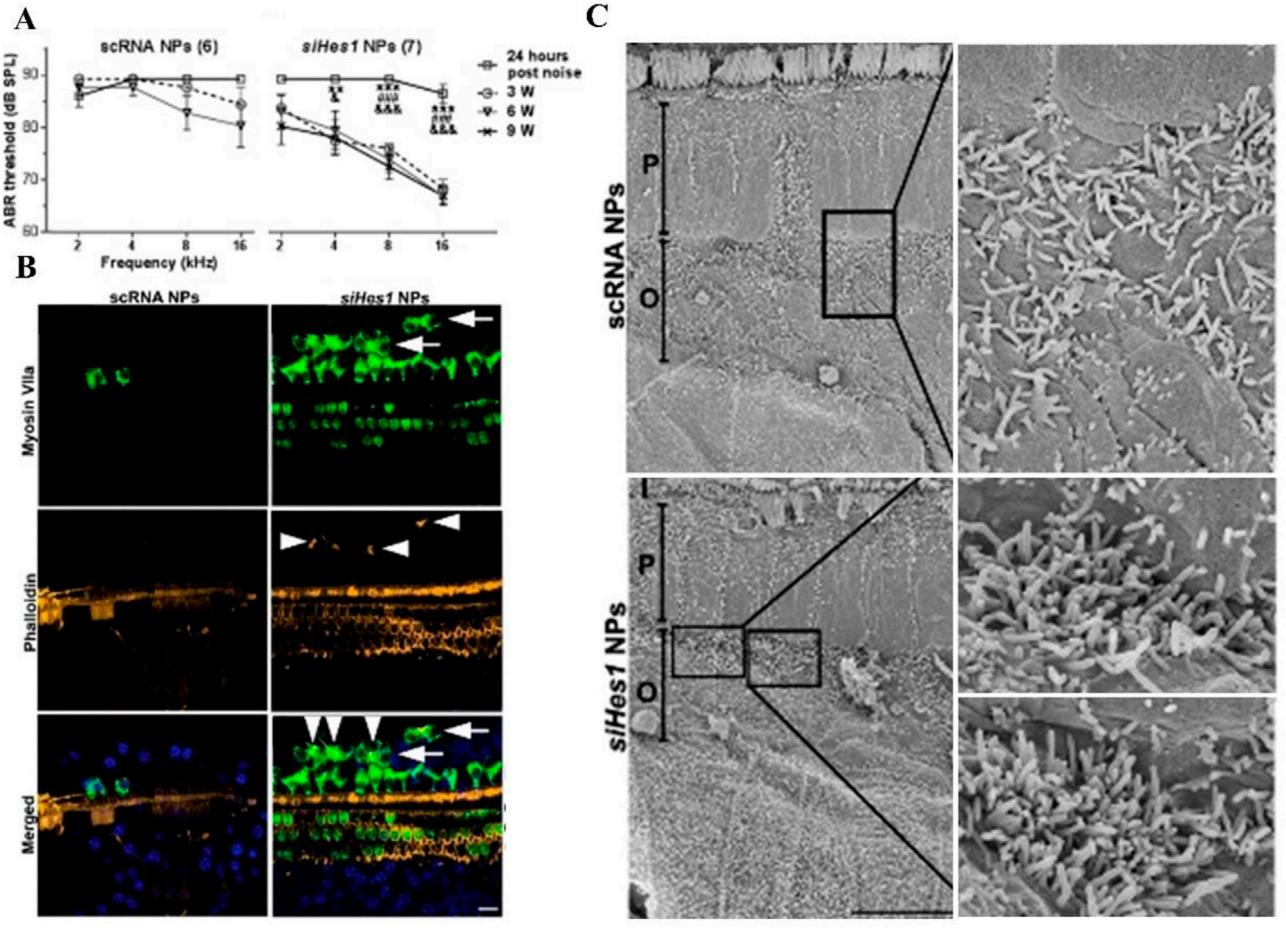
Hair cell restoration and functional recovery were observed only in noise-deafened ears treated with siHes1 encapsulated polylactic/glycolic acid NPs. **(A)** In comparison to pre-treatment ABR thresholds, no significant hearing improvement was observed in noise-deafened guinea pigs treated with scRNA NPs at any time point after treatment. While significant hearing improvement was observed at all time points (3, 6, and 9 weeks after treatment) in siHes1-NP-treated ears across the 4- to 16-kHz frequency range. **(B)** Green, HCs; yellow, stereocilia; blue, 4′, 6-diamidino-2-phenylindole (DAPI) for nuclei. Supernumerary IHCs (arrowheads in green with anti-myosin VIIa) were only observed in siHes1 NP-treated cochleae. **(C)** Clusters of immature HCs with dense and short stereocilia were observed by scanning electron microscopy in noise-deafened cochleae after siHes1 NP treatment. Such cells were not observed in noise-deafened cochleae treated with scRNA NPs. I, P, and O indicate IHCs, pillar cells, and OHCs, respectively.

Du *et al.* delivered biocompatible PLGA NPs containing *Hes1* siRNA payloads into the cochleae of adult guinea pigs *via* unilateral intracochlear infusion by mini-osmotic pumps. This refined pharmacological approach could also induce cochlear hair cell regeneration and hearing recovery in adult animals that had been exposed to a 72-h post-deafening noise trauma (Du et al., 2018) (Figure 5).

A few studies about utilizing nanotechnology to transport biological materials to specific locations to protect damaged spiral ganglion cells (SGCs) have been reported—for example, Zhang *et al.* (Lee et al., 2012) found that delivery of the Tet-1 peptide sequence, which specifically bonded to trisialoganglioside toxin receptors on SGCs and cochlear neurons, can be either through tympanic membrane injection or cochleostomy. The former had no targeted effect in the cochlear ganglion; in contrast, cochleostomy resulted in cochlear nerve targeting.

Meyer et al. (2012) encapsulated rolipram, which has been proven to protect SGC and reduce inflammation, in lipid nanocapsules (LNC). Surprisingly, they found that the combination of rolipram and LNC enabled an increased nerve survival rate, but pure rolipram administration did not. The underlying mechanism was proposed as being not only the synergistic neuroprotective effect of polyethylene glycol (PEG) and rolipram in LNC but also the reduction of intracellular degradation of rolipram encapsulated by LNC. Furthermore, it should be noted that the *in vitro* results have not been confirmed *in vivo* by unilateral cochleostomy experiments, which may be due to the dilution of the perilymph fluid and the inadequate drug release.

In one examination (Martin-Saldana et al., 2017), polymerized NPs containing dexamethasone and α-tocopherol succinate as anti-inflammatory and anti-apoptotic molecules were topically used to treat cisplatin-induced ototoxicity by bullectomy, a cochleostomy, without interfering with the chemical efficacy of cisplatin against cancer. This demonstrated the promising advantage of α-tocopherol succinate NPs to achieve a great therapeutic effect with fewer drug doses and fewer systemic side effects.

Fundamentally speaking, owing to diverse modifiable properties, nanocarriers contain not only a single component but also multi-component systems—the combination of a variety of biomaterials to optimize functions to meet multiple treatment requirements. Advances in hydrogel, including tissue-like mechanical properties and excellent biodegradability, have all sparked interest in the controlled local delivery of drugs and biomaterials to the inner ear using NPs. The combination of the hydrogel system and NP carriers significantly had a more sustained release at the time of administration than the individual NP carriers (Kass and Nguyen, 2021).

### Crossing the Physiological Barriers

The nano-sized particles facilitate drug transport across the physiological barriers in the inner ear due to the smaller size and surface functionalization with the target-specific ligands. Thus, the nano-sized materials can be easily absorbed by the cells and deliver the drug inside.

The authors first deposit ferromagnetic NPs that were administered in phase I human clinical trials systemically into the middle ear by a single intratympanic injection and then magnetically push the particles through the window membranes into the inner ear (Sarwar et al., 2013). Successfully guiding the drug carriers to the target site also requires a good magnet system (Liu et al., 2019).

NPs conjugated with cell-penetrating peptides may be an attractive option to cross protective membranes, such as the RWM, improving transgene expression and the delivery of genes, including DNA or siRNA, into the nuclei of House Ear Institute-Organ of Corti 1 (HEI-OC1) cells (Roy et al., 2010). Arg8-conjugated graft copolymers that were used in gene delivery experiments into inner ear target sites can penetrate the RWM and reach hair cells or SGCs, which are major sites of impairment in cases of hearing loss (Yoon et al., 2015). The nanocarrier-based system showed greater nuclear entry in HEI-OC1 cells and less immunogenicity and toxicity problems than other non-specific PHEA-based particles for application at biomembranes.

Praetorius et al. (2007) discovered that, by the application of modified silica NPs in the round window niche, NPs in the auditory hair cells, vestibular hair cells, and SGNs were seen not only in the treated inner ear but also in the contralateral. Another study demonstrated that disulfiram, a neurotoxic substance (as a toxicity model drug), was used to evaluate the ability of liposomes and polymersomes to enter cochlear cells from the RWM. When adult C3H mice were treated with liposomes and polymersomes without disulfiram for 2 weeks, the average hearing threshold of mice did not change significantly. The number of SGC decreased significantly 2 days after the disulfiram-loaded NP treatment, and the hearing loss was measured by auditory brainstem response (ABR) that reached 20–35 dB 2 weeks post-application. No effect was observed when a blank disulfiram solution was given in the same way. These results show that NPs can effectively transport across physiological membranes in the inner ear and bring biological effects (Buckiova et al., 2012).

Cationic hyperbranched polylysine NPs were detected in both the cytoplasm and nuclei of cochlear cells in primary cell culture, organotypic culture, and *in vivo* by intratympanic administration (Zhang et al., 2011).

### Visualization

As gadolinium serves as an imaging contrast agent, NPs labeled with gadoliniumtetra-azacyclo-dodecane-tetra-acetic acid were applied in the middle ear and analyzed with nuclear magnetic resonance imaging (MRI) to test the drug delivery efficiency of liposome nanocarriers (Zou et al., 2010).

SPIONs are being explored as potential candidates for local delivery by external magnetic fields, tracking the transplanted/injected cells by MRI, and evaluating their engrafting efficiency and functional ability.

The mobility of SPION induced by a magnetic field was also quantified, and the results of flux density, gradients, and NP properties were compared between *in vitro* and *in vivo* models (Barnes et al., 2007). As prospective carriers of therapeutic substances, polymers containing SPION NPs were successfully pulled through both an artificial cochlear RWM model and the guinea pig RWM. Indeed the magnetic force required to pull NPs through the *in vivo* RWM model is significantly lower than that required to achieve in *vitro* transport, suggesting that therapeutic delivery to the inner ear by SPION is feasible.

A pilot study in mice tested the efficacy of the distribution of Cy3-labeled silica NP after placement on the RWM: these NPs were found in the IHCs, OHCs, vestibular hair cells, SGNs, and supporting cells, without any hearing impairment. Since the NP also reached the dorsal cochlear nucleus and the superior olivary complex, the authors suggested a retrograde axonal transport and concluded the safety and feasibility of using silica-based NPs for drug delivery in the auditory (Praetorius et al., 2007).

In earlier studies, the therapeutic effects of systemic or local drug delivery to the inner ear have been discussed by Tamura *et al.* They encapsulated the therapeutic drugs in PLGA NPs and applied them *via* an injection in the femoral vein and RWM, respectively. Analyzing the different fluorescence localization of rhodamine, they found that local drug delivery for rhodamine NPs to the inner ear is more efficient than systemic drug administration (Tamura et al., 2005).

### Improved Biocompatibility and Bioavailability

Hollow MSNs at a zeolitic imidazolate framework capsule also showed good biocompatibility and cellular uptake in HEI-OC1 cells. These results suggest new opportunities to construct a delivery system *via* one controlled low dose and sustained release for the therapy of inner ear disease (Xu et al., 2018). Similarly, the delivery of silica NP-based BDNF as a neuroprotectant to deafened guinea pigs showed a significant improvement in SGN survival after treatment with frusemide and kanamycin (Wang et al., 2014).

In a prospective study by Xu et al. (2018), MSNs which contained gentamicin in the framework of zeolite imidazolyl ester provided a sustained release against Meniere’s disease. Meniere’s disease, a not uncommon clinical syndrome, is characterized by recurrent spontaneous vertigo and tinnitus associated with SNHL. While a lot of pathological studies have been carried out, there are no clear cellular and molecular pathological investigations on the mechanism (Eckhard et al., 2019). MSNs increased the drug loading of gentamicin to 38 wt% and could be taken up into HEI-OC1 cells after subcutaneous injection, which manifested good biocompatibility.

Polyethylene glycol-coated PLGA NPs have been used to carry betamethasone to the cochlea *via* a tail vein injection, which enhanced the therapeutic effect of betamethasone and significantly reduced the cochlear histological and functional damage caused by noise-induced hearing loss. A possible mechanism was proposed by Horie *et al.* who claimed that NPs provided effective and sustained release of the drug in high concentrations and drug accumulation in cochlear tissue (Horie et al., 2010a).

One study by Zou et al. (2010) and colleagues demonstrated that novel gadolinium-labeled liposome NPs and their distribution in the inner ear after intratympanic or intracochlear injection were visualized by MRI. Concerns about the resulting biocompatibility were later supplemented (Zou et al., 2017). In a rabbit model, the intratympanic injection of gadolinium-loaded liposome NPs did not break the biological barrier of the inner ear and activate inflammatory factors, and it also did not change the auditory function as measured by ABR. This shows that the intratympanic injection of liposome NPs *in vivo* is safe.

Besides the liposome NPs mentioned above, mesoporous silica possesses high porosity, large specific surface area, and drug loading, which sustainedly release and provided sufficient drug concentration in local treatment. It is worth noting that Lensing et al. (2013) showed that the local release of ciprofloxacin from the middle ear prosthesis coated with nanoporous silica layer could restrain the proliferation of bacteria and prevent an infection caused by the middle ear prosthesis.

NP delivery uses a chitosan–glycerophosphate (CGP) hydrogel system (nanohydrogel) to bring therapeutic biomaterials to specific cellular structures within the inner ear (Lajud et al., 2013). The system consisted of CGP hydrogel loaded with the drug that was placed directly on the round window niche and which, at body temperature, became an adherent semi-solid gel that persisted in the niche. Hydrogels help retain drugs for sustained release. The use of chitosanase to digest the CGP–hydrogel makes the drug loaded in the system no longer in contact with the RWM, which could then be washed away through the Eustachian tube and effectively discontinued from entering the inner ear when symptoms disappear or when adverse side effects first appear.

Nanohydrogel may offer a noninvasive and sustained solution to deliver therapeutic agents, which is crucial for clinical application. Studies on dexamethasone phosphate (DexP) loading within liposomes and their stability during the storage delivery systems have reported promising preliminary results. *In vivo* experiments confirmed that the HA-DexP-Lip gel has no negative effect on the hearing function. Furthermore, they have shown that liposomal NPs delivered *via* nanohydrogel persisted under physiologic conditions for up to 30 days, thus demonstrating sustained release and degradation capability (El Kechai et al., 2016).

### Challenge and Opportunity

The development of nanocarriers and nano-based delivery systems has enabled hearing to be restored in mouse models. Unfortunately, major defects such as low transfection efficiency as a gene carrier still needs to be overcome, while adeno-associated viruses (AAVs), which have minimal pathogenic and immunogenic effects, have proven that the technique applied to the cochlea is relatively safe, highly efficacious, and targeted to date (Colella et al., 2018). The AAV-2 serotype (AAV2/1, 2/2, 2/5, 2/7, 2/8, and 2/9) displays tropism for hair cells and supporting cells (Bedrosian et al., 2006; Konishi et al., 2008; Akil et al., 2012). However, one major disadvantage of AAVs is their size. Only a small cDNA (up to 4.7 kb) can be packaged using an AAV without a risk of dysfunction, which may require splitting the deafness genes into two or three parts to avoid exceeding the packaging capacity of AAV (Akil, 2020). Therefore, non-viral delivery offers a powerful complementary approach for delivering therapeutic agents to the inner ear.

As nonviral vectors, nanocarriers have their obvious advantages, such as the ability to transfer a gene cassette of unlimited size and type and the absence of a viral component that may evoke an immunogenic and inflammatory response. Polyethylenimine is often regarded as the criterion standard in nonviral gene delivery *in vitro* due to strong DNA compaction capacity and endosomal escape (Tan et al., 2008). It can be used successfully to mediate cochlear gene transfer, displaying improved transfection efficiency with sustained release of the vector solution. Polyamine did show an expression of GFP in the area of the organ of Corti and the inner hair cell (Praetorius et al., 2008), while the combination of a non-liposomal lipid with a DNA-condensing component did not show transfection of the organ of Corti.

In addition, these nanovectors are not like lentivirus, which may integrate into a host genome despite effective antiretroviral therapy. Therefore, they may minimize the risk of insertional mutagenesis and infections persisting in another site. Maeda *et al.* identified that the *GJB2*-targeting siRNA was encapsulated in a liposome, resulting in selectively suppressing the *GJB2*_R75W_ expression and preventing hearing loss. The level of endogenous murine Gjb2 expression in mice was not affected (Maeda et al., 2005).

Besides these, these nanovectors are easy to manufacture, potentially safer for delivering drugs in humans, and more flexible. Drugs and genes have all been successfully encapsulated in NPs to prolong the time during which they are protected against enzymatic destruction. In the exciting development for gene therapies, a very important field of medical research has approved gene delivery based on nanocarriers: patisiran, which has improved outcomes in intracranial malignancy (Zou et al., 2020; Wang et al., 2021). The NPs can integrate temozolomide (TMZ), a chemotherapy drug for treating glioblastomas with small interfering RNAs to suppress the TMZ resistance gene (MGMT) from binding specifically to tumor cells, significantly prolonging survival as compared to mice treated with TMZ alone.

In summary, knowledge about cationic polymer transfection reagents and the underlying mechanisms contributing to functional delivery have grown and substantially increased. However, critical problems that are correlated to their molecular weight remain, leading to a certain degree of cytotoxicity upon *in vivo* administration. Another limitation of cationic polymers in the inner ear is the lesser transfection efficiency for outer hair cells, resulting in only a partial restoration of hearing. Therefore, polymer systems should be generated with improved precision and reduced cytotoxicity.

## Tuning Nanomedicine Efficacy

The processes of nanomedicine delivery system into the inner ear, such as biological diffusion, distribution, cellular uptake, and elimination and metabolism from plasma to tissue, can be tuned by multiple nanomaterial physicochemical properties.

### Size

The specific properties of numerous nanomaterials in the treatment of ear diseases are dependent on size. In many cases, smaller-sized NPs exhibit a better activity than the larger ones, given that NPs with a smaller size have a higher surface-to-volume ratio, which is helpful for passing through the biofilm barrier. The RWM is not only a drug delivery channel but also a selective barrier. Most studies have suggested that small-molecular-weight and nanometer-scale particles can pass through the RWM, but those of a few microns cannot (Goycoolea et al., 1988). The effectiveness of liposome NPs, with the size of 95, 130, and 240 nm, in crossing the middle ear barrier in Wister rats was compared. The larger the liposome NPs were, the lesser the number of liposome NPs from the middle ear to the inner ear was (Zou et al., 2012). Conversely, some studies found that a NP with a larger size probably behaves better than a smaller one. Comparing 80-, 150-, and 300-nm Coumarin-6-loaded NPs, it was found that the 150- and 300-nm NPs could penetrate the RWM into the cochlea faster than the 80-nm NPs. This conclusion may come from the complexity of the transport mechanism of NPs into the inner ear (Cai et al., 2017). The endocytosis pathway, as a way for liposome NPs to pass through the RWM, varies depending on the size of the endocytosis (Conner and Schmid, 2003). The result showed that the size of the liposome carrier is an important factor that affects the passage from the middle ear to the inner ear. In other words, NPs with different sizes have different endocytosis pathways, resulting in different diffusivity through the RWM. In addition, the clearance levels of NPs of different sizes is different by tympanic injection through the Eustachian tube.

### Surface Modification

The physical properties of many nanomaterials for treating inner ear diseases, such as surface charge, hydrophilicity or hydrophobicity, and solubility, can affect as to whether the drug can be dissolved in perilymph through the RWM and eventually absorbed by inner ear cells. There are negatively charged glycoproteins on the surface of the cell membrane, so positively charged NPs could help to improve the uptake by cells (Lee et al., 2012). It is reported that positively charged NPs carry drugs to the cochlea, in contrast to negative and neutral ones (Cai et al., 2017). Moreover, monoglyceride NPs with a high surface charge, especially a positive charge, showed a more significant uptake in the RWM and more widespread distribution in the cochlea (Liu et al., 2013). Surprisingly, Yang *et al.* found that, although the uptake ability of positive NPs was enhanced among neutral, negative, and positive phospholipid NPs, they may be attached to cells and extracellular matrix and have difficulty in penetrating the RWM of the inner ear (Yang et al., 2018). The NPs on the hydrophilic surface are distributed in the cochlea fastest (Cai et al., 2017), which may suggest that the hydrophilic-molecule-modified nano-based drug delivery system has a bright prospect for catering to clinical inner ear diseases.

### Forming Hybrids

The hydrogels mentioned above can prolong the retention time of loading drugs but often release drugs too quickly. While NPs can control the release, reduce the degradation, and allow drugs to be targeted to the inner ear, a hybrid system, like these two materials, can regulate the pharmacokinetics of drugs delivered to the inner ear. An example of a hybrid delivery platform is the combination of hyaluronic acid gel and liposome. The hyaluronic acid gel increased the retention time of nanocarriers in the middle ear and increased the concentration of adrenocortical hormone in the inner ear, while liposomes sustained drug release in the perilymph for up to 30 days and promoted the conversion of dexamethasone phosphate to dexamethasone. By tracking rhodamine-labeled liposomes after a middle ear injection, it was shown that only a small amount of NPs appeared in the perilymph of guinea pigs, and most of them were blocked in the round window of guinea pigs (El Kechai et al., 2016).

Another example of a hybrid system is the application of PLGA NPs and chitosan gel. On the one hand, chitosan gel provides a friendly environment for protein peptides and increases the contact time with the RWM. On the other hand, PLGA NPs containing interferon α-2b (IFN α-2b) reduce drug clearance. The combination of these two drug delivery systems improves the efficacy of polypeptide drug delivery in the inner ear (Dai et al., 2018). Using the hybrid of certain drug delivery systems with tunable properties may be a potential strategy to overcome the deficiency of the individual use of each delivery system.

## Conclusion and Prospect

For decades, great efforts have been devoted to developing an effective, non-invasive, sustainable drug delivery system to stabilize the treatment of biomaterials and target-specific parts of the inner ear. These advances in nanotechnology provide effective and non-invasive tools for the delivery of drugs and biomaterials. Up to now, nano-carriers such as polymersome, liposome, silica NP, and phospholipid NP can successfully carry a large drug through the RWM into the inner ear as shown in an *in vitro* experiment, especially integrating with hydrogels to complement their respective shortcomings. In addition, some specific properties and other modified properties of nanomaterials can be employed to optimize the efficacy of various inner ear diseases in the future. Superparamagnetic iron oxide NPs are magnetic, and their motion is controlled by an external magnetic field. Besides this, there are also gadolinium-containing liposome NPs, whose distribution in organisms can be observed by MRI. It is worth mentioning that the application of animal models to cure specific inner ear diseases *in vivo* is limited, such as for autoimmune ear diseases. Therefore, before successfully translating the experimental results into clinical application, some problems need to be solved and more *in vivo* studies need to be carried out to ensure their effectiveness and safety. In addition to traditional therapy, as the potential for gene therapy in the treatment of many diseases becomes evident in study directions, advancements in gene therapy technologies will help the translation of these discoveries to be achieved. Despite many successful investigations, definitive data on the application of gene therapy to inner ear diseases are lacking to date, and more research may be needed to develop safer and more efficient nanocarriers for inner ear disease gene therapy.

## Abbreviations

PLGA, polylactic/glycolic acid.
